# Prevalence of Micronutrient Deficiencies and Relationship with Clinical and Patient-Related Outcomes in Pulmonary Hypertension Types I and IV

**DOI:** 10.3390/nu13113923

**Published:** 2021-11-01

**Authors:** Paulien Vinke, Thomas Koudstaal, Femke Muskens, Annemien van den Bosch, Michiel Balvers, Mieke Poland, Renger F. Witkamp, Klaske van Norren, Karin A. Boomars

**Affiliations:** 1 Nutritional Biology, Division of Human Nutrition and Health, Wageningen University, Stippeneng 4, 6708 WE Wageningen, The Netherlands; michiel.balvers@wur.nl (M.B.); mieke.poland@wur.nl (M.P.); renger.witkamp@wur.nl (R.F.W.); klaske.vannorren@wur.nl (K.v.N.); 2 Department of Pulmonary Medicine, Erasmus MC, University Medical Center Rotterdam, Dr. Molewaterplein 40, 3015 GD Rotterdam, The Netherlands; t.koudstaal.1@erasmusmc.nl (T.K.); a.m.muskens@erasmusmc.nl (F.M.); a.e.vandenbosch@erasmusmc.nl (A.v.d.B.); k.boomars@erasmusmc.nl (K.A.B.)

**Keywords:** pulmonary hypertension, pulmonary arterial hypertension, chronic thrombo-embolic pulmonary hypertension, vitamin D, iron, micronutrient deficiencies

## Abstract

Background: Pulmonary hypertension (PH) is a rare progressive and lethal disease affecting pulmonary arteries and heart function. The disease may compromise the nutritional status of the patient, which impairs their physical performance. This study aimed to determine the prevalence of micronutrient deficiencies in pulmonary arterial hypertension (PAH) and chronic thrombo-embolic pulmonary hypertension (CTEPH) patients. Methods: Eighty-one blood samples from a prospective observational cohort study were analyzed for concentrations of micronutrients and inflammation-related factors. The samples consisted of newly diagnosed (treatment-naive) PAH and CTEPH patients and patients treated for 1.5 years according to ERS/ESC guidelines. Results: In the newly diagnosed group, 42% of PAH patients and 21% of CTEPH patients were iron deficient compared to 29% of PAH patients and 20% of CTEPH patients in the treatment group. Vitamin D deficiency occurred in 42% of the newly diagnosed PAH patients, 71% of the newly diagnosed CTEPH patients, 68% of the treated PAH patients, and 70% of the treated CTEPH patients. Iron levels correlated with the 6 min walking distance (6MWD). Conclusions: Iron and vitamin D deficiencies are highly prevalent in PAH and CTEPH patients, underlining the need for monitoring their status. Studies evaluating the effects of supplementation strategies for iron and vitamin D are necessary.

## 1. Introduction

Pulmonary hypertension (PH) refers to a heterogeneous group of diseases characterized by vasoconstriction and structural remodeling of the pulmonary arteries. Increased vascular resistance and elevated pulmonary arterial pressures lead to right ventricular hypertrophy and eventually to heart failure and premature death [1,2]. Symptoms are progressive and include inflammation, right ventricular dysfunction, serious exercise intolerance, and fatigue, reducing the quality of life of the patients [3,4]. Recently, the role of inflammatory processes in different types of PH has gained more attention [5,6,7,8,9,10]. PH is divided into five subgroups according to the classification of the World Health Organization (WHO), European Respiratory Society (ERS), and European Society of Cardiology (ESC) [11]. In this study, we focused on patients with WHO type 1: pulmonary arterial hypertension (PAH) and type 4: chronic thrombo-embolic pulmonary hypertension (CTEPH), because these patients are typically treated with PH-specific therapies in expert centers. In both categories, the pathophysiology originates in the pulmonary arteries. The prognosis of both PAH and CTEPH is poor, with 3- and 5-year survival rates for PAH of 67% and 57–59%, respectively [11,12,13] and 5-year survival rates of 53–59% for inoperable CTEPH [13,14,15]. The prognosis of CTEPH patients with central lesions who are eligible for and who underwent pulmonary endarterectomy is improved, with 5-years survival rates of around 85% [16].

The ESC/ERS guidelines for pulmonary hypertension contain various recommendations for PH treatment, although there are few on nutrition. Moreover, there are no data on the influence of nutritional status on prognostic indicators, as defined by the ESC/ERS [11]. However, nutritional status, including the occurrence of micronutrient deficiencies, is likely to be relevant to both the overall physiological status and the exercise capacity of the patient. It is known that micronutrient deficiencies, such as those of magnesium, vitamin D, and iron, are linked to inflammatory processes, which may contribute to exercise intolerance [17,18]. Vice versa, a reduction in physical activity can have consequences for vitamin D synthesis and might also affect dietary habits. Hence, monitoring and optimization of nutritional status in patients with PH hold promise to improve the overall well-being of PH patients. Literature research reveals a lack of studies on nutritional deficiencies in PAH and CTEPH patients and identifies important knowledge gaps regarding the potential benefits of nutritional intervention on the quality of life of these patients [3]. It has been suggested that anemia and iron deficiency are prevalent in CTEPH and in PAH patients in particular [19]. As with other individuals, these factors are likely to have a negative impact on exercise tolerance and fatigue in PH [11,20]. Compared to iron, even less is known of the status of other relevant micronutrients, including vitamin D and magnesium in PH patients. Although magnesium status is relevant for cardiac and skeletal muscle function, glucose homeostasis [21,22], and chronic inflammation [23], it is not routinely monitored. Vitamin D deficiency is highly prevalent in Europe during the winter season and in chronically ill patients or the elderly also throughout the year [18]. Deficiency of vitamin D is linked to bone health, muscle dysfunction, and attenuated immune function [18,24]. However, inflammation itself can impact vitamin D status, for example, via decreasing vitamin-D-binding protein (DBP), leading to lower 25-hydroxy-vitamin D (25(OH)D) levels in the blood [25]. So far, there is limited evidence of the potential benefit of correcting vitamin D deficiency in patients with PAH. In summary, studies into the prevalence of micronutrient deficiencies and their effects on clinical outcomes in PAH and CTEPH patients are currently lacking, although these deficiencies can negatively influence the patient’s health status. This would ultimately merit suppletion strategies, although more research would be needed on this. 

This study aimed to determine the actual status of micronutrients that may play a role in inflammation, fatigue, and exercise intolerance in PAH and CTEPH patients at the time of diagnosis and after 1.5 years of conventional treatment. A secondary aim was to explore whether there is a correlation between micronutrient deficiencies and clinical outcome parameters. Although pulmonary arterial hypertension is relatively rare, this study collected data on micronutrient status and many clinical and patient-related outcome variables of a relatively large group of patients from the Biopulse prospective observational cohort study.

## 2. Materials and Methods

### 2.1. Patients and Study Design

A prospective observational cohort study was conducted in the Erasmus University Medical Center for specialized PH care since May 2012. Pulmonary hypertension patients >18 years old with a mean pulmonary arterial pressure (mPAP) of ≥25 mmHg, a wedge pressure of ≤15 mmHg, and a pulmonary vascular resistance (PVR) of ≥3WU measured by right heart catheterization (RHC) at diagnosis were invited to take part in the study. Diagnosis of PAH and CTEPH patients was made according to the ERS/ECSC guidelines [2,11] and subclassification according to the WHO classification system. Patient characteristics and PH subgroups are shown in Table 1 and Table 2 in the Results section. The newly diagnosed group consisted of 33 treatment-naive PAH and CTEPH patients. The treated group was a different group of 48 PAH and CTEPH patients who received conventional PH-specific treatment for 1.5 years. The treated group was therefore not a follow-up of the newly diagnosed group. Exclusion criteria were incomplete diagnostic work-up and therefore no confirmed PH diagnosis, age <18 years, or not being capable of understanding or signing informed consent. The study protocol was approved by the medical ethical committee (MEC-2011-392). Written informed consent was provided by all patients. The study was performed in accordance with the principles of the Declaration of Helsinki.

### 2.2. Clinical Data Collection

Baseline data were collected during the inpatient screening visit. At baseline, all patients underwent physical examination by a pulmonary physician and a cardiologist, 6 min walking test (6MWT), pulmonary function tests, VQ scan, chest computed tomography scan, ultrasound of the liver, 12-lead electrocardiography (ECG), echocardiography, venous blood sampling, and RHC. Patient characteristics and vital signs collected at baseline included age, sex, height, weight, systemic blood pressure, heart rate, and peripheral oxygen saturation. The NYHA functional class was used to grade the severity of functional limitations. During RHC, a Swan–Ganz catheter was inserted in the internal jugular vein. A standardized protocol for the work-up of PH was used to obtain hemodynamic measurements. Fick’s principle was used to measure cardiac output. If the obtained capillary wedge pressure was ambiguous, a fluid challenge was performed to distinguish pre-capillary PH from PH due to left heart disease. Data were collected and stored in PAHTool (version 4.3.5947.29411; Inovoltus, Santa Maria da Feira, Portugal), an online electronic case report form.

### 2.3. Clinical Follow-Up

Patients were treated according to the ERS/ESC guidelines [11]. CTEPH patients were assessed for eligibility for either pulmonary endarterectomy or balloon pulmonary angioplasty. Patients who underwent one of the above procedures were not censored afterward. All patients were prospectively followed up by half-yearly scheduled visits to the outpatient clinic. During this visit, vital signs were collected, the NYHA functional class was graded, venous blood samples were taken (e.g., for measurement of N-terminal prohormone brain natriuretic peptide (NT-pro-BNP)), and a 6MWT as well as echocardiography were performed.

### 2.4. Venous Blood Sampling

Venous blood samples were obtained at baseline and every 6 months during follow-up. Transfer of the blood samples to the clinical chemistry laboratory occurred within 2 h from withdrawal. Hemoglobin (Hb) and NT-pro-BNP were directly determined in the fresh blood samples. Serum samples and plasma samples were processed and aliquoted and stored at −80 °C until further analysis.

### 2.5. Laboratory Analyses

Measurements of plasma iron (ferrum), ferritin, transferrin, magnesium, calcium, phosphate, vitamin B12 (cobalamin), and folic acid were performed in serum samples according to standard operating procedures of the clinical chemistry lab of the Erasmus University Medical Center, Rotterdam. Vitamin D was analyzed using the Fujirebio Lumipulse G1200 system with a Lumipulse 25-OH Vitamin D assay (Fujirebio, Tokyo, Japan), catalog nos. 234020 for calibrators and 234013 for reagents) according to the manufacturer’s instructions. All other analytes were determined using a Cobas 8000 system (Roche, Rotkreuz, Switzerland). The concentrations of each micronutrient for individual patients were compared with specific reference values, as validated by the Erasmus University Medical Center, Rotterdam, and as specified in Table 3, Table 4 and Table 5. The reference value for vitamin D was specified as >50 nmol/L [26]. Patients with concentrations below the reference value were scored as having a too low circulating concentration of that micronutrient. 

Hepcidin values were analyzed using a human hepcidin (Hepc) ELISA kit (Cusabio, catalog no. CSB-E16062h) in undiluted EDTA plasma samples according to the manufacturer’s protocol. Vitamin-D-binding protein was analyzed using a GC-Globulin (vitamin-D-binding protein) Human ELISA kit (Abcam, catalog no. Ab108853, Cambridge, UK) in undiluted EDTA plasma samples according to the manufacturer’s protocol. Reference values for vitamin-D-binding protein and hepcidin were specified according to the ELISA kits’ manufacturers’ manuals.

### 2.6. Statistics

The unpaired, two-tailed *t*-test in Graphpad Prism (version 5, San Diego, CA, USA) was performed to compare groups. Data were presented as the mean + SD. Missing data were not replaced in the analysis. Spearman’s correlation was performed in Rstudio using the ggscatter function. Significance was accepted as *p* < 0.05.

## 3. Results

### 3.1. Characteristics of Participants

In this study, 33 newly diagnosed patients with PAH and CTEPH and 48 patients who were under conventional PH-specific treatment for 1.5 years (treated group) were included. In the newly diagnosed group, 19 patients were diagnosed with PAH and 14 patients with CTEPH. Baseline characteristics did not differ between persons diagnosed with PAH or CTEPH, except for height (Table 1). There were no differences in age, weight, body mass index (BMI), 6 min walking distance (6MWD), right ventricular systolic pressure (RVSP) measured by echocardiography, and mean PAP and cardiac index (CI) at RHC. As shown in Table 2, in the treated group, 38 patients with PAH and 10 patients with CTEPH were included. The mean age of the CTEPH patients was higher than the age of the PAH patients (64.5 versus 48.8 years, respectively). This is not surprising since in general, PAH patients tend to be younger than CTEPH patients. No differences were observed in height, weight, and BMI between patients with PAH and CTEPH in this group. The percentage of females in each group was relatively similar (63% and 70%, respectively). The 6MWD was not different between patients with PAH or CTEPH, both at baseline and after 1.5 years of treatment. The baseline characteristics for the RVSP, mean PAP, PVR, and CI are shown in Table 2 for each patient group, as well as the RVSP after 1.5 years of treatment. 

### 3.2. Prevalence of Abnormal Micronutrient and Mineral Blood Concentrations 

The percentage of patients with levels below the reference value for the micronutrients analyzed in both patient groups with the diagnostic subclasses are shown in Table 3 and Table 4. The levels of micronutrients and nutrient-related factors in all newly diagnosed and treated patients can be found in Supplemental Figures S1–S5.

#### 3.2.1. PAH Patients

In newly diagnosed patients with PAH, 42% could be classified as deficient in 25(OH)D (60% of the males and 36% of the females). Moreover, 21% of newly diagnosed PAH patients had a too low circulating concentration of magnesium. 

In the group under treatment for 1.5 years, 68% of the patients with PAH had 25(OH)D levels below normal (79% of the males and 63% of the females) and 16% had a too low circulating concentration of phosphate. 

#### 3.2.2. CTEPH Patients 

In newly diagnosed patients with CTEPH, 71% of the patients had 25(OH)D levels below the reference value (63% of the males and 83% of the females). Moreover, 14% showed a low circulating concentration of calcium and 7% had a low circulating concentration of magnesium. 

Of the CTEPH patients after 1.5 years of treatment, 70% had a too low 25(OH)D level (100% of the males and 57% of the females), 20% had a low circulating concentration of phosphate, and 10% showed a low circulating concentration of magnesium. 

#### 3.2.3. Newly Diagnosed versus Treated Patients

Of all newly diagnosed patients in our study, 15% had a low circulating concentration of calcium, another 15% had a low circulating concentration of magnesium, and 55% of the patients showed a low 25(OH)D concentration. Of all the treated patients, 69% showed too low 25(OH)D levels. Furthermore, a total of 17% of the treated patients had a too low circulating concentration of phosphate.

#### 3.2.4. Female Patients

Of all the newly diagnosed female patients in our study, 50% had too low 25(OH)D levels, 20% had a low circulating concentration of magnesium, and 15% had a low circulating concentration of calcium.

Of all female patients in the treated group, 61% had a too low 25(OH)D level and 13% had a too low circulating concentration of phosphate.

#### 3.2.5. Male Patients

A total of 62% of the newly diagnosed males had a low 25(OH)D concentration, 16% had a low circulating concentration of calcium, and 8% had a low circulating concentration of magnesium.

After 1.5 years of treatment, 82% of the males had a too low 25(OH)D level and 24% had a low circulating concentration of phosphate.

#### 3.2.6. Summary

Overall, a large part of the patients in our study were found to be deficient in 25(OH)D, both at diagnosis as well as after 1.5 years of treatment. At diagnosis, vitamin D deficiency was more prevalent in CTEPH patients than in PAH patients. In the treated group, the prevalence of vitamin D deficiency was similar in both PAH and CTEPH patients. Furthermore, low circulating concentrations for magnesium, calcium, and phosphate were demonstrated. 

### 3.3. Iron Status in Newly Diagnosed and Treated PH Patients 

Table 5 presents the values for the analytes specifically related to iron status. 

In the newly diagnosed group, 30% of the females and 8% of the males had too low Hb levels and could therefore be classified as having anemia. In addition, 33% of the newly diagnosed patients had a low circulating concentration of plasma iron. Of all the newly diagnosed PAH patients, 42% had low circulating iron concentrations compared to 21% of the newly diagnosed CTEPH patients. In addition, 31% of the newly diagnosed males and 40% of the females had a too low transferrin saturation.

In the group under treatment for 1.5 years, 26% of the females and 24% of the males had too low Hb levels and could therefore be classified as having anemia. In this group, 27% of the patients had a low circulating concentration of plasma iron. Of all the PAH patients after 1.5 years of treatment, 29% had a low circulating concentration of iron compared to 20% of the treated CTEPH patients. A total of 59% of the treated males and 36% of the females had low transferrin saturation. In summary, a considerable number of the patients were deficient in iron, especially female patients. Iron deficiency was higher in PAH patients than in CTEPH patients, both at the time of diagnosis as well as after 1.5 years of treatment.

### 3.4. Correlations between Micronutrient Deficiencies and Clinical Outcome

**Figure 1:**
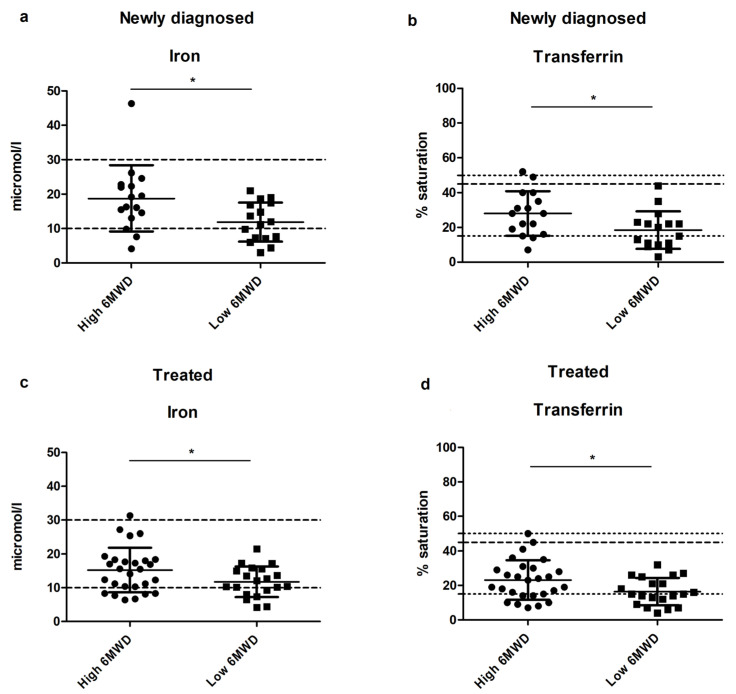
Iron levels and transferrin saturation in newly diagnosed and treated patients with a higher or lower 6MWD than the group mean (* *p* < 0.05). (**a**) Iron levels in newly diagnosed PAH and CTEPH patients with a high vs. low 6MWD. (**b**) Transferrin saturation levels in newly diagnosed PAH and CTEPH patients with a high vs. low 6MWD. (**c**) Iron levels in treated PAH and CTEPH patients with a high vs. low 6MWD. (**d**) Transferrin saturation levels in treated PAH and CTEPH patients with a high vs. low 6MWD. 6MWD: six-minute walk distance, PAH: pulmonary arterial hypertension, CTEPH: chronic thrombo-embolic pulmonary hypertension.

A significant positive correlation was found between the 6MWD and plasma iron levels and between the 6MWD and transferrin saturation in both newly diagnosed and treated patients (all *p* < 0.01); see Supplemental Figure S6. By looking at the treated group with a result of the 6MWD below and above the group mean (439 m), we observed that both iron and transferrin saturation levels were significantly lower in the group with a 6MWD below the mean compared to the group with a 6MWD above the mean (both *p* < 0.05); see Figure 1a,b. NT-pro-BNP levels of the group with a 6MWD below the mean were significantly higher than those of the group with a 6MWD above the mean (with means of 104.6 pg/mL and 22.15 pg/mL, respectively; *p* < 0.05); see Supplemental Figure S7. In the newly diagnosed group, both plasma iron and transferrin saturation levels were also significantly lower in the group with a 6MWD below the mean (both *p*< 0.05); see Figure 1c,d. NT-pro-BNP levels were not significantly different in the newly diagnosed group between those with a 6MWD below the mean compared with those with a 6MWD above the mean (see Supplemental Figure S7). Other micronutrients tested were not significantly different between groups with a 6MWD below or above the mean. This was the case in both newly diagnosed and treated groups. 

**Figure 2:**
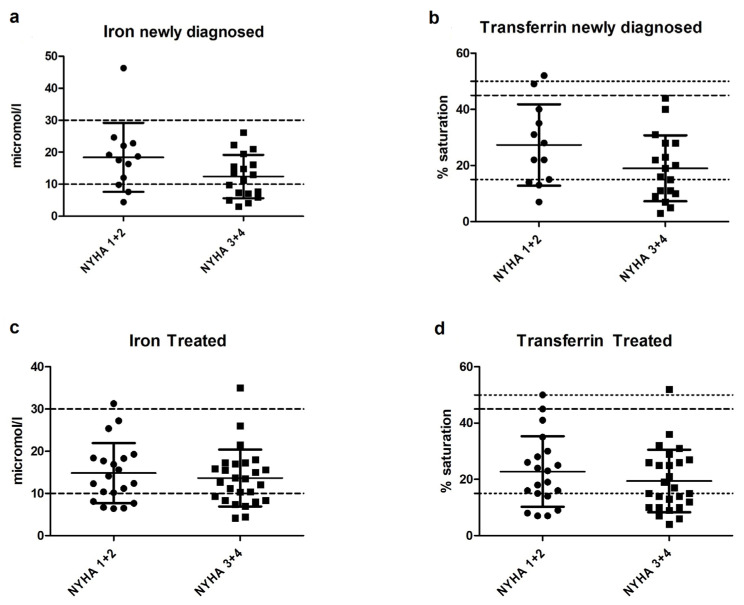
Iron levels and transferrin saturation in newly diagnosed and treated patients with NYHA classes 1 and 2 versus NYHA classes 3 and 4. (**a**) Iron levels in newly diagnosed PAH and CTEPH patients with NYHA classes 1 and 2 vs. NYHA classes 3 and 4. (**b**) Transferrin saturation levels in newly diagnosed PAH and CTEPH patients with NYHA classes 1 and 2 vs. NYHA classes 3 and 4. (**c**) Iron levels in treated PAH and CTEPH patients with NYHA classes 1 and 2 vs. NYHA classes 3 and 4. (**d**) Transferrin saturation levels in treated PAH and CTEPH patients with NYHA classes 1 and 2 vs. NYHA classes 3 and 4. NYHA: New York Heart Association.

With respect to the NYHA class, a trend toward higher iron and higher transferrin saturation levels in the lower NYHA classes (1 and 2) versus the higher NYHA classes (3 and 4) was observed in the newly diagnosed patients but not in the treated patients (see Figure 2). The mean plasma iron level in newly diagnosed patients was 18.43 μmol/L in NYHA classes 1 and 2 and 12.41 μmol/L in NYHA classes 3 and 4 (*p* = 0.07). The mean transferrin saturation in newly diagnosed patients was 27.3% in NYHA classes 1 and 2 compared to 19.0% in NYHA classes 3 and 4 (*p* = 0.09). Levels of the other micronutrients tested were not different between groups with lower and higher NYHA classes. This was the case in both the newly diagnosed and the treated group.

### 3.5. The Relation between Iron, Hepcidin, and Inflammation

**Figure 3:**
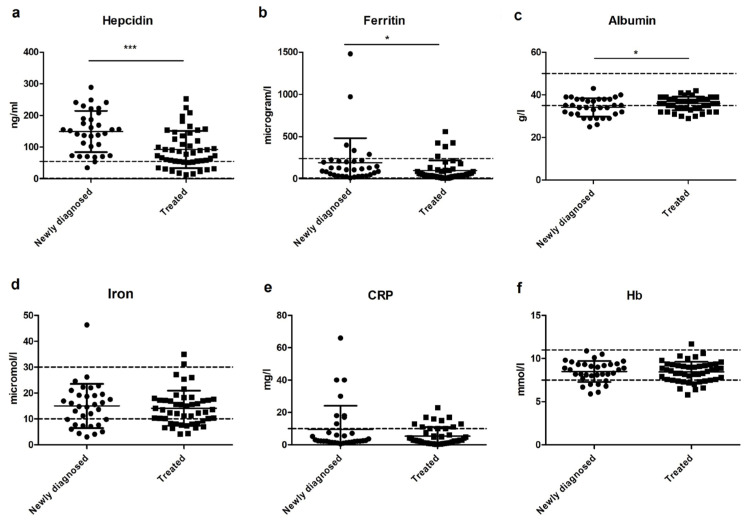
Iron, hepcidin, and inflammation levels in newly diagnosed and treated patients (* *p* < 0.05, *** *p* < 0.001). (**a**) Hepcidin levels were higher in newly diagnosed than treated patients with PAH or CTEPH (*p* < 0.001). (**b**) Ferritin levels were higher in newly diagnosed than treated patients with PAH or CTEPH (*p* < 0.05). (**c**) Albumin levels were lower in newly diagnosed than treated patients with PAH or CTEPH (*p* < 0.05). (**d**) Iron levels in newly diagnosed and treated patients with PAH or CTEPH were not different. (**e**) CRP levels in newly diagnosed and treated patients with PAH or CTEPH were not different. (**f**) Hb levels in newly diagnosed and treated patients with PAH or CTEPH were not different.

By evaluating the results on iron status and inflammation in all patients, it becomes clear that hepcidin levels were higher in newly diagnosed patients compared with treated patients (*p* = 0.0001; see Figure 3a) and ferritin levels as well (*p* < 0.05; see Figure 3b). Albumin levels were significantly lower in the newly diagnosed group compared with the treated group (*p* < 0.05; see Figure 3c). This suggests that inflammation might play a role. Circulating levels of plasma iron, C-reactive protein (CRP), and Hb levels were not significantly different between the newly diagnosed and treated groups (see Figure 3d–f). 

Considering the hepcidin levels of specific patients with low circulating iron concentration, relatively high values were observed in the newly diagnosed group (mean hepcidin level: 121 g/L; mean iron level: 6.53 μmol/L). At the same time, these levels were low in patients with a low circulating iron concentration in the treated group (mean hepcidin level: 57 g/L; mean iron level: 7.10 μmol /L). 

### 3.6. Vitamin-D-Binding Protein and 25(OH)D

**Figure 4:**
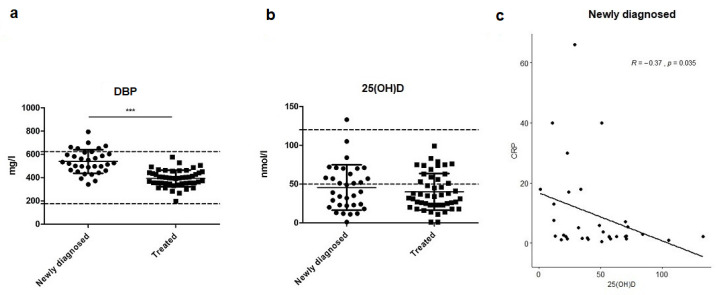
Levels of 25(OH)D and vitamin-D-binding protein in newly diagnosed and treated patients (*** *p* < 0.0001). (**a**) Levels of vitamin-D-binding protein (DBP) were reduced in treated patients compared with newly diagnosed patients with PAH or CTEPH. (**b**) 25(OH)D levels were not different in newly diagnosed versus treated patients with PAH or CTEPH. (**c**) There was a negative correlation between the levels of 25(OH)D and CRP in newly diagnosed patients.

The levels of DBP were strongly reduced in treated patients compared with newly diagnosed patients (mean values 393.4 mg/L and 538.6 mg/L, respectively; *p* < 0.0001; see Figure 4a). In general, lower DBP levels can lead to lower 25(OH)D levels measured in plasma. However, in our patients, 25(OH)D levels were not statistically different in treated patients compared to the levels in newly diagnosed patients (mean values 40.0 nmol/L and 45.5 nmol/L, respectively; see Figure 4b). A significant negative correlation was found between 25(OH)D and CRP levels in newly diagnosed patients (R = −0.37, *p* < 0.05; see Figure 4c) but not in treated patients. There was no significant correlation between 25(OH)D and albumin levels.

## 4. Discussion

The nutritional status in patients with PAH and CTEPH is an understudied field, although recent papers suggest that nutritional status and micronutrient deficiencies are likely to influence the quality of life of these patients via their potential effects on inflammatory status, skeletal muscle functions, and other (patho-)physiological processes [3,27]. Moreover, hypoxia is a common symptom in PAH patients and is associated with disease progression [28]. In COPD patients, there is a relationship between hypoxia and decreased mitochondrial function in skeletal and respiratory muscles [29,30]. Hypoxia might have a negative effect on mitochondrial function in the skeletal and respiratory muscles of PAH patients as well, contributing to fatigue. Increased fatigue can negatively influence nutritional intake and so impact micronutrient status. However, this hypothesis will have to be studied in the future.

Our study is the first showing the micronutrient status in both newly diagnosed as well as treated patients with PAH and CTEPH. Deficiencies in iron and vitamin D are highly prevalent in patients with PAH and CTEPH, both at diagnosis and after 1.5 years of conventional treatment. Depending on the diagnostic subclass, iron deficiency occurs in up to 42% of the patients and vitamin D deficiency in up to 83% of the patients. The 25(OH)D status even seems to worsen during the disease course. Iron deficiency is more prevalent in female than in male patients and is also more prevalent in patients with PAH than in patients with CTEPH. Other micronutrient deficiencies are observed for magnesium and phosphate, with prevalence rates up to 21%. Lower iron levels and transferrin saturation are linked to a lower 6MWD in both groups at diagnosis and after 1.5 years of treatment. 

### 4.1. Iron Deficiency

This study showed that 42.1% of the newly diagnosed PAH patients and 21.4% of the newly diagnosed CTEPH patients had low circulating levels of plasma iron. In the treated group, this was 28.9% for PAH patients and 20% for CTEPH patients. In addition, in the newly diagnosed group, 30% of the females and 8% of the males had a too low Hb level. In the treated group, 2.8% of the females and 2.5% of the males had a too low Hb level, indicating anemia. An earlier study reported a prevalence of iron deficiency of 43% in idiopathic PAH patients. Moreover, these authors found that the 6MWD is reduced in iron-deficient patients compared with iron-sufficient patients irrespective of the existence of anemia [20]. They also demonstrated that intravenous iron therapy in 15 iron-deficient iPAH patients improved exercise endurance capacity, but not the 6MWD [31]. 

This study showed that there is a correlation between iron levels, transferrin saturation, and the 6MWD. The levels of circulating iron and transferrin saturation are significantly lower in patients with a 6MWD lower than the average compared with patients with a higher 6MWD. 

In addition, our study showed, in agreement with other studies [19,20], that the prevalence of anemia or iron deficiency is higher in patients with PAH than in patients with CTEPH. This has been suggested to be related to interleukin-6 (IL-6) levels, because IL-6 levels correlate with iron levels in idiopathic PAH patients but not in CTEPH patients [19]. Inhibition of dietary iron uptake by the negative regulator of plasma iron levels, hepcidin, might be causally related to this effect. The main iron transporter of the intestine (ferroportin) is inhibited by hepcidin, resulting in decreased intestinal absorption of iron [31,32,33]. Inflammatory cytokines, such as interleukin 1-beta (IL-1β) and IL-6, stimulate hepcidin transcription via different mechanisms, leading to excessive hepcidin production. Interleukin 1-beta might even provide the onset signal, as it induces the transcription of IL-6. This process is also being referred to as inflammation-induced anemia [34]. 

In this study, increased inflammation was shown by lower albumin levels in the newly diagnosed group and was also associated with increased hepcidin levels. Albumin is a negative acute-phase protein, meaning that plasma levels go down in inflammatory conditions [35]. Hepcidin values found in our study were above the range normally seen in healthy people. However, it should be noted that it is unknown which hepcidin values can be expected in chronically ill patients. The physiological challenge for these patients is how to maintain adequate iron stores. Oral supplementation with the recommended daily dose might not be useful when hepcidin levels are high due to underlying inflammation [33]. In this situation, intravenous iron supplementation might be preferable. A recent study found that oral iron supplementation with ferric maltol was well tolerated in 20 patients with PH and iron deficiency anemia and restored iron and hemoglobin levels [36]. This type of oral iron supplementation might therefore be an alternative for intravenous iron supplementation in PH patients. 

Taken together, it is currently unknown whether low iron levels can be prevented by supplementation with the normal recommended daily dose of oral iron in combination with anti-inflammatory treatment. Future studies are needed to reveal this. Moreover, the effect of the use of anti-inflammatory drugs on iron status in PH patients should also be studied.

### 4.2. Vitamin D Deficiency

Vitamin D deficiency is related to increased inflammation and deregulation of the immune system in several chronic diseases [37]. In our patient group, vitamin D was the most prevalent micronutrient deficiency, and this deficiency persists during the disease course. Depending on the therapeutic subclass, vitamin D deficiency occurs in 40–83% of the patients. In the normal European population, vitamin D deficiency has a prevalence of 40%, when taking vitamin D levels of <50 nmol/L as a cutoff and of 13% at cutoff levels of <30 nmol/L [38,39]. Another study examined the difference between vitamin D deficiency (25–50 nmol/L) and vitamin D insufficiency (50–75 nmol/L) and found a prevalence of 41% and 33%, respectively [40]. A lower DBP level was found in the treated group compared to the newly diagnosed group, although there was no difference in 25(OH)D. It is possible that the treated group had higher levels of free vitamin D. In this study, total vitamin D was measured: free and bound vitamin D. Furthermore, we did not find a correlation between 25(OH)D levels and outcome parameters. Therefore, it remains unclear what exactly the significance of vitamin D deficiency is in patients with PH. 

The question whether supplementation of vitamin D would be clinically useful in patients with PH is dependent on many factors, such as body fat and lifestyle. It has been found that the active elderly who spend much time outdoors have a low risk of vitamin D deficiency [41]. Inactivity and remaining indoors can therefore increase the risk of vitamin D deficiency in PH patients. A large European study found that seasonally adjusted 25(OH)D concentrations are lower in smokers and higher with increasing ultraviolet B (UVB) exposure, dietary vitamin D or oily fish consumption, and supplement use with omega-3 fatty acids and vitamin D [40]. Moreover, having a high BMI or being overweight is associated with lower vitamin D status or response to supplementation, which is explained by volumetric dilution and/or sequestration in the adipose tissue [42,43,44]. There is limited scientific knowledge on vitamin D supplementation to increase vitamin D status in patients with PH. An uncontrolled longitudinal study supplemented 22 PH patients with 50,000 IU cholecalciferol weekly plus a preparation providing 200 mg magnesium, 8 mg zinc, and 400 IU vitamin D daily for 3 months. Serum 25(OH)D and 6MWD significantly increased in this study [45]. In another study, vitamin D supplementation increased serum vitamin D levels in a rat model of PH [46]. There is, however, convincing evidence that vitamin D supplementation is effective to increase 25(OH)D levels to an optimal level in the general population [38]. More studies are needed to establish the effect of vitamin D supplementation on its status and clinical outcome in PH patients. However, there are recommendations for vitamin D supplementation in the healthy general population that can be used for patients with PH until disease-specific recommendations can be given [26].

### 4.3. Summary and Recommendations

In summary, the prevalence of vitamin D and iron deficiency is high in patients with PAH and CTEPH. Therefore, it is recommended to monitor iron and vitamin D status in these patients. In the case of deficiency, supplementation of iron and vitamin D should be considered. For iron, oral supplementation may not be successful in the case of underlying inflammation. In these cases, intravenous supplementation might work. The effect of vitamin D supplementation in PH patients needs further evaluation.

## 5. Conclusions

Both iron and vitamin D deficiencies were highly prevalent in the two groups of PAH and CTEPH patients at the time of diagnosis and after 1.5 years of conventional treatment. Vitamin D deficiency was the most prevalent micronutrient deficiency in these patient groups. There was no difference between the newly diagnosed and the treatment group. Iron deficiency occurred more often in patients with PAH than in patients with CTEPH and more often in females than in males. Moreover, data suggested that low iron levels negatively impact exercise tolerance. Therefore, this study shows the need for monitoring micronutrient status, especially for iron and vitamin D, in PAH and CTEPH patients. To treat these deficiencies in PAH and CTEPH patients, future studies are needed to investigate the effect of different supplementation strategies both for iron and for vitamin D.

## Figures and Tables

**Table 1:** Characteristics of patients in the newly diagnosed group.

Characteristic	PAH	CTEPH
*n* (%)	19 (58)	14 (42)
Gender, female (%)	14 (74)	6 (43)
Age, year	62.5 ± 14.7	63.9 ± 12.9
Height, cm	164.6 ± 9.8	174.5 ± 10.0 *
Weight, kg	72.9 ± 18.7	80.1 ± 17.6
BMI, kg/m^2^	26.7 ± 5.6	26.4 ± 5.9
NYHA class (1:2:3:4)	0:6:12:1	2:5:6:1
NT-pro-BNP, pmol/L **	351 ± 863	224 ± 232
Cause of pulmonary hypertension		
HPAH (%)	2 (11)	
IPAH (%)	4 (21)	
CTD (%)	9 (47)	
CHD (%)	0 (0)	
Portopulmonary (%)	3 (16)	
Drugs/toxins/medication (%)	1 (5)	
Other (%)	0 (0)	
Type of CTD-related PAH		
SSc (%)	7 (78)	
SLE (%)	1 (11)	
Sjogren (%)	1 (11)	
6MWD, m	352 ± 121	447 ± 240
Echocardiography, RVSP in mm/Hg	76.5 ± 19.9	72.1 ± 21.0
R-catheterization		
Mean PAH, mmHg	46.5 ± 14.0	42.0 ± 11.9
PAWP, mmHg	9.7 ± 3.6	9.3 ± 4.3
PVR, WU	7.3 ± 3.3	6.2 ± 3.2
CI	2.7 ± 0.7	2.8 ± 0.8

All patients tested HIV negative. * *p* < 0.05, ** *p* < 0.01 compared to the PAH group. ** NT-pro-BNP: PAH median: 62 and IQR: 222; CTEPH: median: 180 and IQR: 315. PAH: pulmonary arterial hypertension, CTEPH: chronic thrombo-embolic pulmonary hypertension, BMI: body mass index, NYHA: New York Heart Association, NT-pro-BNP: N-terminal pro-brain natriuretic peptide, HPAH: heritable pulmonary arterial hypertension, IPAH: idiopathic pulmonary arterial hypertension; CTD: connective tissue disorders, CHD: coronary heart disease, SSc: systemic sclerosis, SLE: systemic lupus erythematosus, 6MWD: six-minute walk distance, RVSP: right ventricular systolic pressure, PAWP: pulmonary artery wedge pressure, PVR: pulmonary vascular resistance, WU: wood units, CI: cardiac index.

**Table 2:** Characteristics of patients in the treated group.

Characteristic	PAH	CTEPH
*n* (%)	38 (79)	10 (21)
Gender, female (%)	24 (63)	7 (70)
Age, year	48.8 ± 15.1	64.5 ± 12.0 **
Height, cm	168.7 ± 10.8	169.8 ± 6.3
Weight, kg	75.6 ± 18.4	83.3 ± 11.3
BMI, kg/m^2^	26.7 ± 7.1	28.9 ± 3.9
NYHA class (1:2:3:4)	1:16:19:2	2:3:5:0
NT-pro-BNP, pmol/L **	71 ± 157	23 ± 20
Cause of pulmonary hypertension		
HPAH (%)	2 (5)	
IPAH (%)	14 (37)	
CTD (%)	9 (24)	
CHD (%)	8 (21)	
Portopulmonary (%)	4 (11)	
Drugs/toxins/medication (%)	0 (0)	
Other (%)	1 (3)	
Type of CTD-related PAH		
SSc (%)	7 (78)	
SLE (%)	2 (22)	
Sjogren (%)	0 (0)	
PAH-specific drugs		
PDE-5 inhibitor (%)	33	4
ERA (%)	33	8
sGCs (%)	1	1
Prostacycline receptor agonist (Selexipag) (%)	5	0
Prostacycline (IV) (%)	6	0
Treprostinil (IV/SC) (%)	2	0
Drug combination therapy		
Monotherapy (%)	5	3
Duo therapy (%)	21	5
Triple therapy (%)	11	0
6MWD, m		
Baseline	374 ± 140	346 ± 99
18 months follow-up	445 ± 142	420 ± 121
Echocardiography, RVSP in mmHg		
Baseline	76.9 ± 18.6	61.5 ± 17.5 *
18-month Follow-up	62.7 ± 22.6	48.1 ± 17.9
R-catheterization (baseline)		
Mean PAP, mmHg	51.3 ± 13.8	38.9 ± 13.5 *
PAWP, mmHg	9.8 ± 3.3	9.8 ± 3.5
PVR, WU	9.6 ± 4.9	5.1 ± 2.6 *
CI	2.6 ± 0.7	2.7 ± 0.2

All patients tested HIV negative. * *p* < 0.05, ** *p* < 0.01 compared to the PAH group. ** NT-pro-BNP: PAH median: 26 and IQR: 34; CTEPH median: 17 and IQR: 16.

**Table 3:** Percentage of patients below the reference value for micronutrients in newly diagnosed and treated patients, split by gender and disease classification.

	Newly Diagnosed					Treated				
	Total	Female	Male	PAH	CTEPH	Total	Female	Male	PAH	CTEPH
	(*n* = 33)	(*n* = 20)	(*n* = 13)	(*n* = 19)	(*n* = 14)	(*n* = 48)	(*n* = 31)	(*n* = 17)	(*n* = 38)	(*n* = 10)
Iron (<10 µmol/L)	33%	40%	23%	42%	21%	27%	36%	12%	29%	20%
Magnesium (<0.7 mmol/L)	15%	20%	8%	21%	7%	4%	3%	6%	3%	10%
Calcium (<2.2 mmol/L)	15%	15%	15%	16%	14%	2%	0%	6%	3%	0%
Phosphate (<0.8 mmol/L)	0%	0%	0%	0%	0%	17%	13%	24%	16%	20%
Vitamin B11 (<5 nmol/L)	0%	0%	0%	0%	0%	0%	0%	0%	0%	0%
Vitamin B12 (<145 pmol/L)	3%	5%	0%	5%	0%	4%	7%	0%	5%	0%
25(OH)D (<50 nmol/L)	55%	50%	62%	42%	71%	69%	61%	82%	68%	70%

**Table 4:** Percentage of patients below the reference value for micronutrients in newly diagnosed and treated in gender subgroups of PAH and CTEPH patients.

	Newly Diagnosed				Treated			
	PAH Males	PAH Females	CTEPH Males	CTEPH Females	PAH Males	PAH Females	CTEPH Males	CTEPH Females
	(*n* = 5)	(*n* = 14)	(*n* = 8)	(*n* = 6)	(*n* = 14)	(*n* = 24)	(*n* = 3)	(*n* = 7)
Iron (<10 µmol/L)	20%	50%	25%	17%	14%	38%	0%	29%
Magnesium (<0.7 mmol/L)	0%	29%	13%	0%	0	4%	33%	0%
Calcium (<2.2 mmol/L)	20%	14%	13%	17%	7%	0%	0%	0%
Phosphate (<0.8 mmol/L)	0%	0%	0%	0%	29%	14%	0%	29%
Vitamin B11 (<5 nmol/L)	0%	0%	0%	0%	0%	0%	0%	0%
Vitamin B12 (<145 pmol/L)	0%	7%	0%	0%	0%	8,3%	0%	0%
25(OH)D (<50 nmol/L)	60%	36%	63%	83%	79%	63%	100%	57%

**Table 5:** Percentage of patients below the reference value for iron-related factors in newly diagnosed and treated patients, split by gender and disease classification.

	Newly Diagnosed					Treated				
	Total	Female	Male	PAH	CTEPH	Total	Female	Male	PAH	CTEPH
	(*n* = 33)	(*n* = 20)	(*n* = 13)	(*n* = 19)	(*n* = 14)	(*n* = 48)	(*n* = 31)	(*n* = 17)	(*n* = 38)	(*n* = 10)
Hb (F: <7.5 mmol/L, M: <8.5 mmol/L)	21%	30%	8%	26%	14%	25%	26%	24%	24%	30%
Iron (<10 mmol/L)	33%	40%	23%	42%	21%	27%	36%	12%	29%	20%
Ferritin (F: <10 µg/L, M: <30 µg/L)	3%	0%	8%	0%	7%	6%	0%	18%	8%	0%
Transferrin sat. (F: <15%, M: <20%)	36%	40%	31%	47%	21%	44%	36%	59%	47%	30%

## Data Availability

Data were collected and stored in PAHTool (version 4.3.5947.29411; Inovoltus, Santa Maria da Feira, Portugal), an online electronic case report form.

## Supplementary Materials

The following are available online at https://www.mdpi.com/article/10.3390/nu13113923/s1, Figure S1: Micronutrient levels of newly diagnosed and treated patients, Figure S2: Hemoglobin levels of newly diagnosed and treated male and female patients, Figure S3: Ferritin levels of newly diagnosed and treated male and female patients, Figure S4: Levels of magnesium, calcium, and phosphate in newly diagnosed and treated patients, Figure S5: Levels of vitamin B12, folic acid, and vitamin D in newly diagnosed and treated patients, Figure S6: Correlation of the 6MWD with iron and transferrin saturation levels, Figure S7: NT-pro-BNP levels of newly diagnosed and treated patients with a 6MWD below and above the mean.
