# Supplementary material for: Prevalence of Micronutrient Deficiencies and Relationship with Clinical and Patient-Related Outcomes in Pulmonary Hypertension Types I and IV
Source: Nutrients. 2021 Nov 1;13(11):3923. doi: 10.3390/nu13113923 (PMC8617670; doi:10.3390/nu13113923)

Prevalence of micronutrient deficiencies and relation with clinical and patient-related outcomes in pulmonary hypertension type I and IV

Author names: Paulien Vinke [1], Thomas Koudstaal [2], Femke Muskens [2], Annemien van den Bosch [2], Michiel Balvers [1], Mieke Poland [1], Renger F Witkamp [1], Klaske van Norren [1#], Karin A Boomars [2#]

Author affiliations:

1. Nutritional Biology, Division of Human Nutrition and Health, Wageningen University, Stippeneng 4, 6708 WE Wageningen, the Netherlands

2. Department of Pulmonary Medicine, Erasmus MC, University Medical Center Rotterdam, Dr. Molewaterplein 40, 3015 GD Rotterdam, The Netherlands

#: contributed equally

Correspondence to: Paulien Vinke, Division of Human Nutrition and Health, Nutritional Biology Group, Wageningen University, Wageningen, the Netherlands. Stippeneng 4, 6708 WE Wageningen, The Netherlands. E-mail: paulien.vinke@posteo.de. ORCID iD: 0000-0001-6657-8065.

Supplemental data

Figure S1: Micronutrient levels of newly diagnosed and treated patients

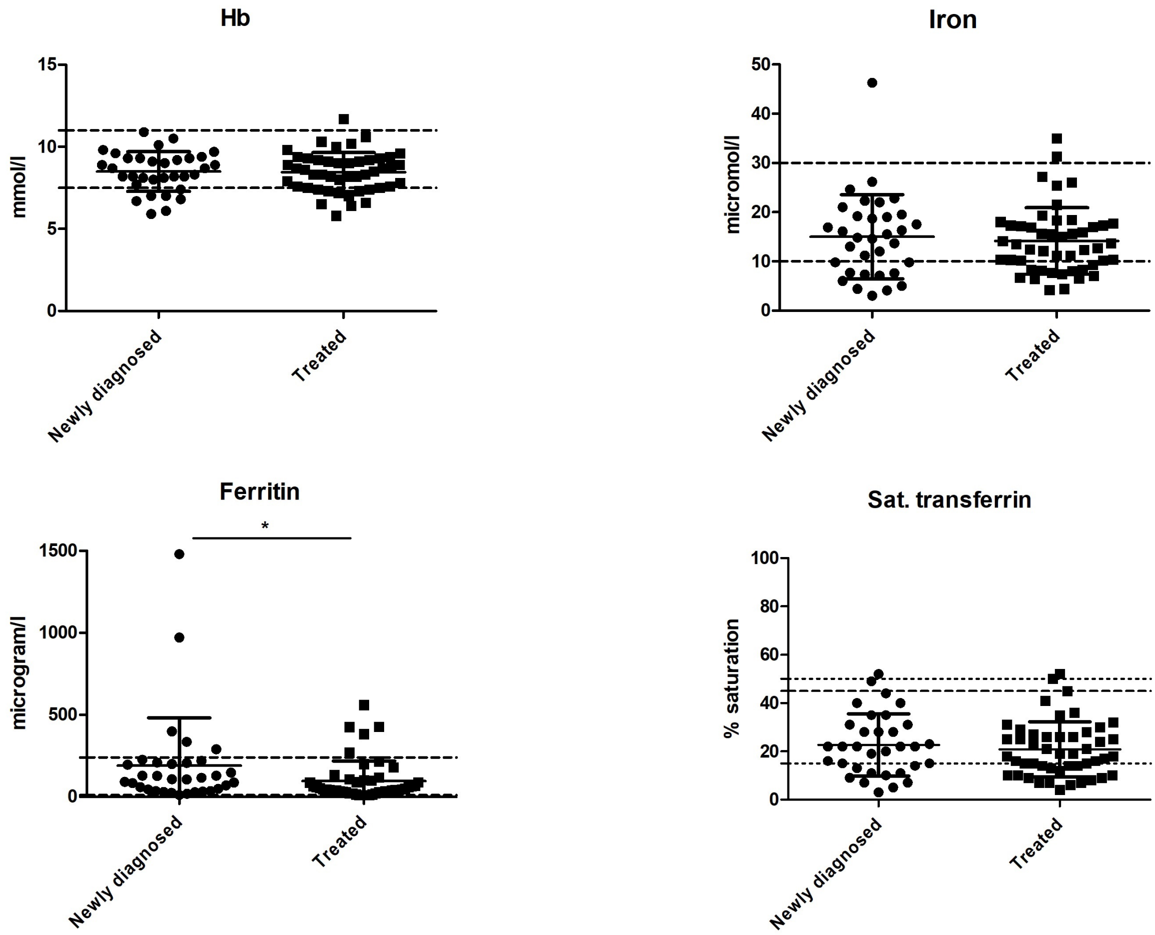

Figure S2: Hemoglobin levels of newly diagnosed and treated male and female patients

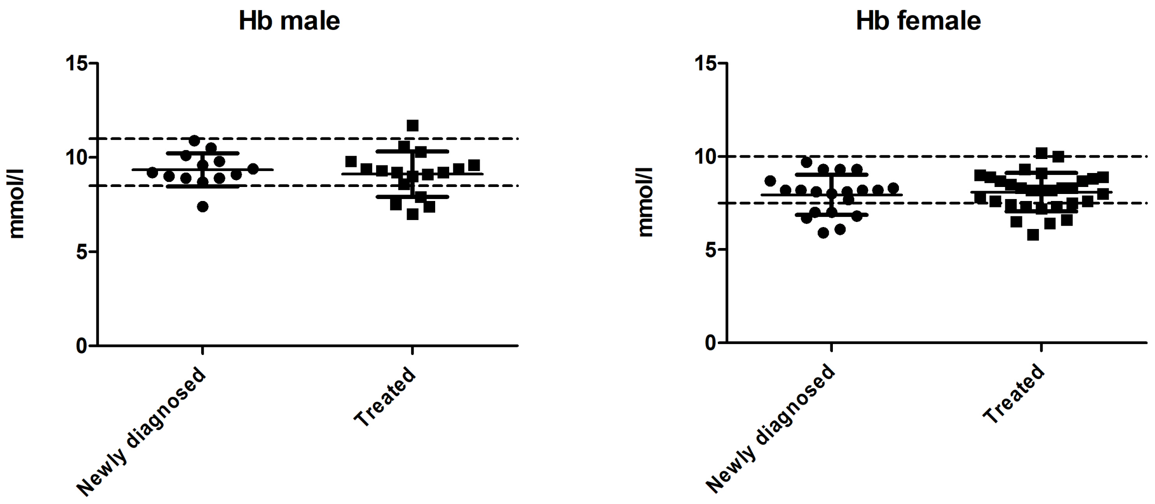

Figure S3: Ferritin levels of newly diagnosed and treated male and female patients

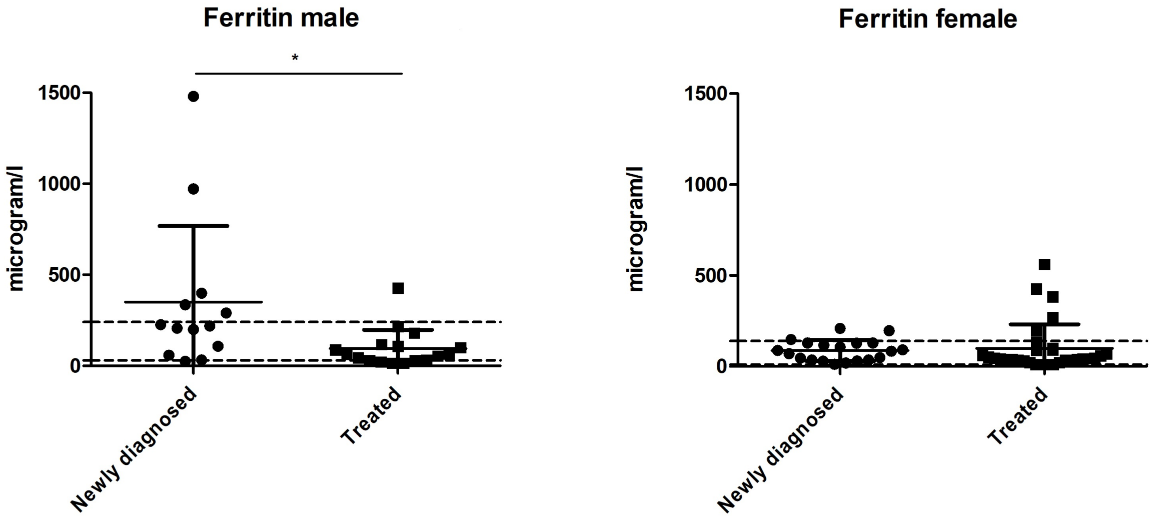

Figure S4: Levels of magnesium, calcium and phosphate in newly diagnosed and treated patients

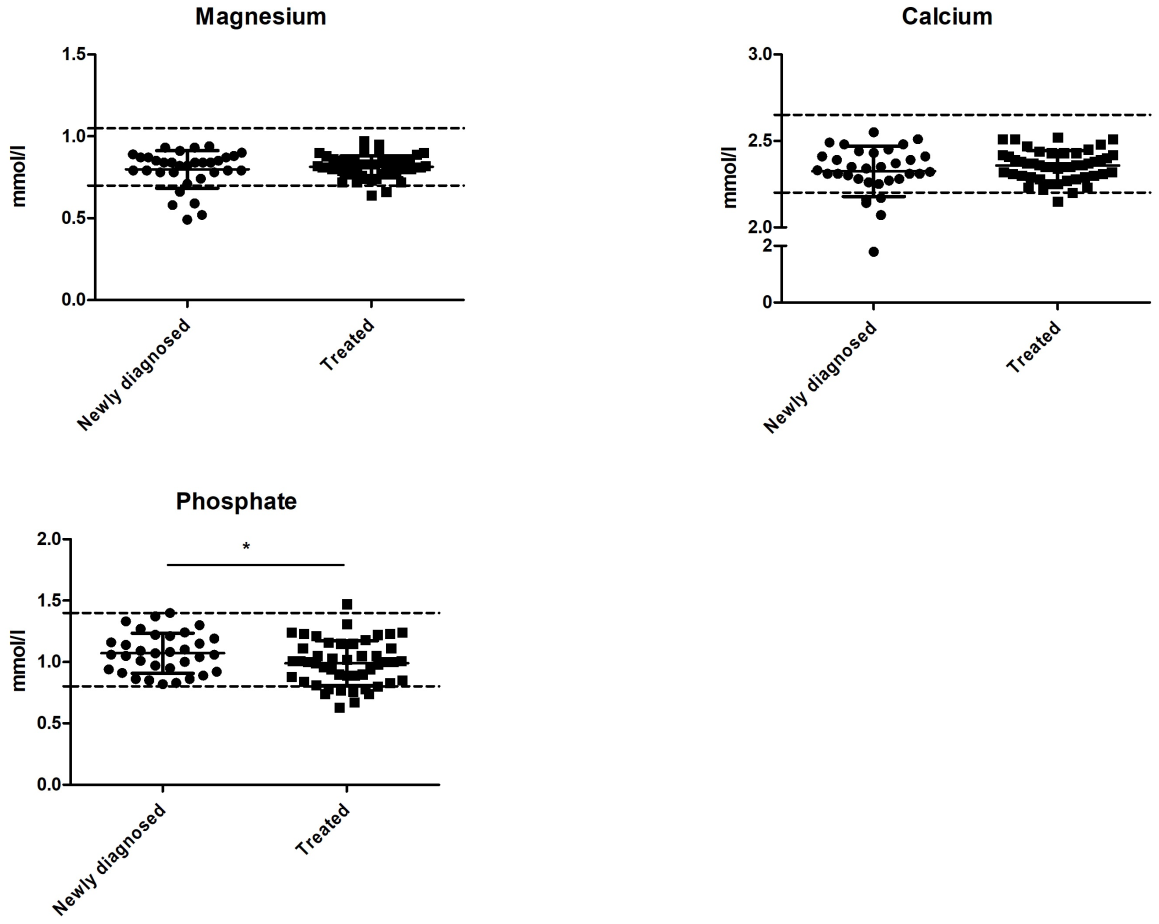

Figure S5: Levels of vitamin B12, folic acid and vitamin D in newly diagnosed and treated patients

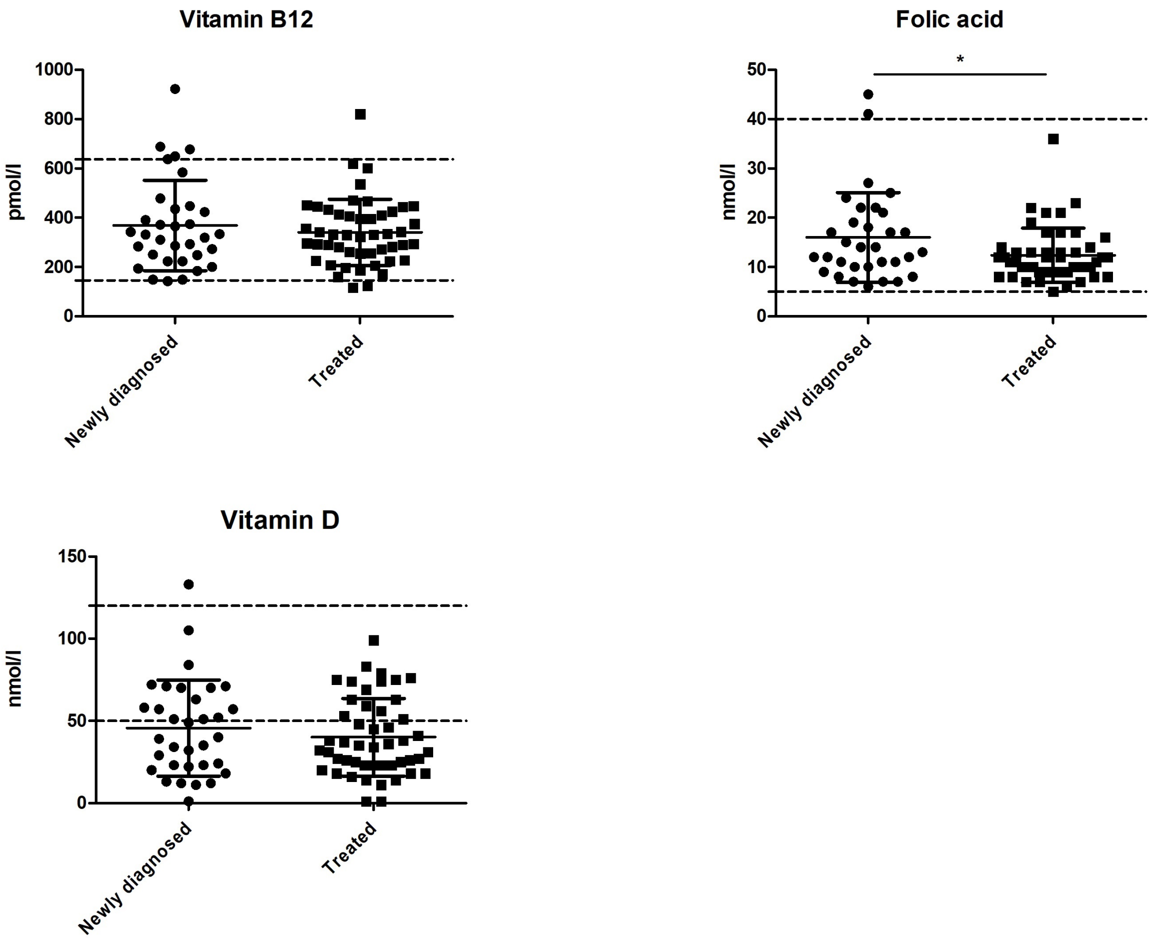

Figure S6: Correlation 6MWD with iron and transferrin saturation levels

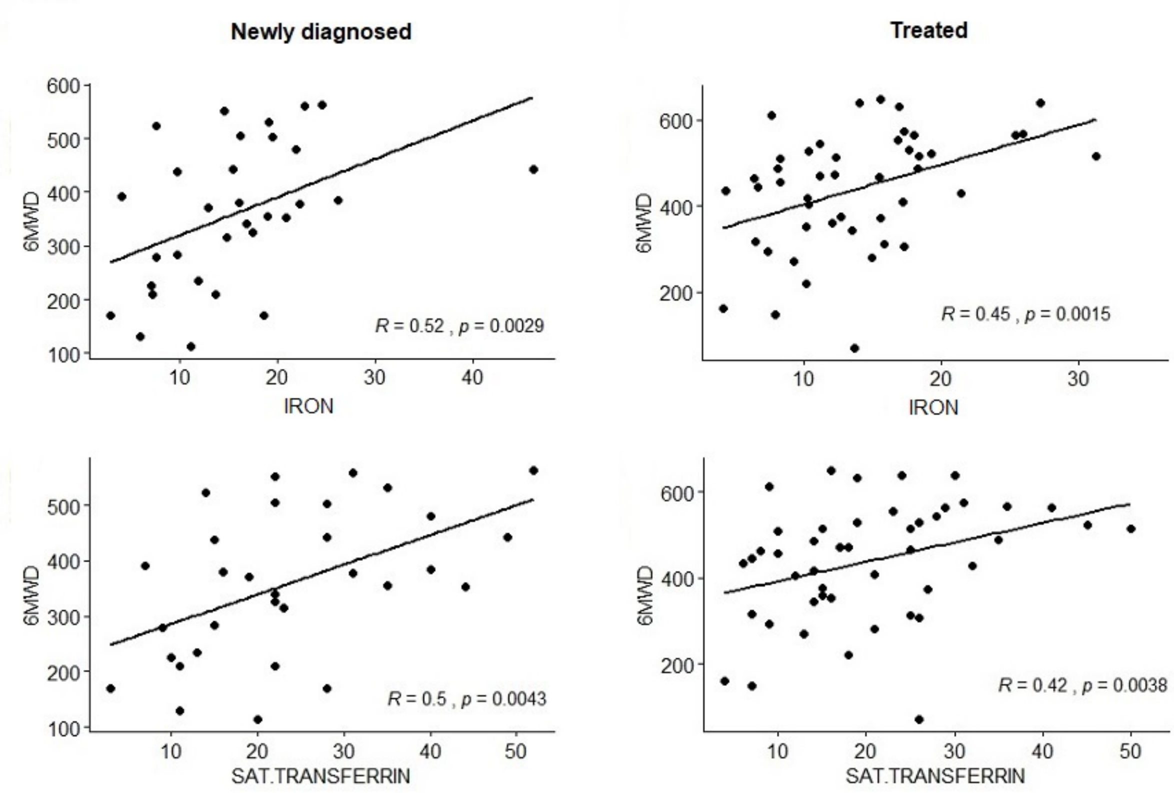

Figure S7: NT-Pro-BNP levels of newly diagnosed and treated patients with 6MWD below and above the mean.

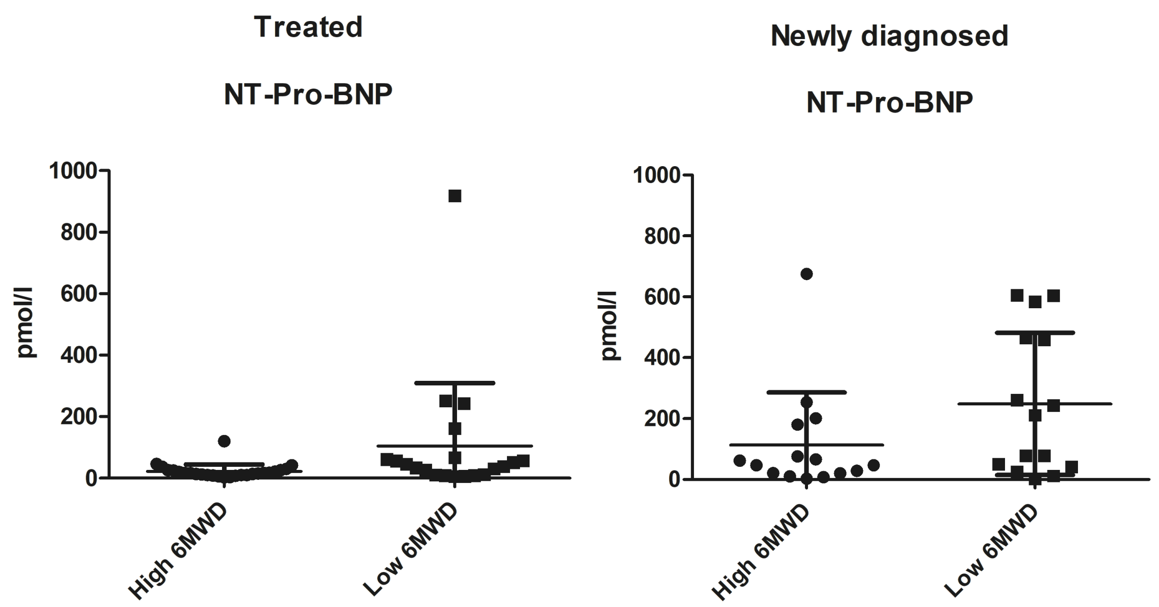

Supplement: Supplementary file 1 [file nutrients-13-03923-s001.zip › nutrients-1404230-supplementary.pdf]
